# Avaliação técnica do dispositivo de fechamento vascular Exoseal-Cordis^®^

**DOI:** 10.1590/1677-5449.000717

**Published:** 2017

**Authors:** Altino Ono Moraes, Rogério Yoshikazu Nabeshima, Ericsson Fernando Viotto, Marcelo Hiroshi Estevam Yoshida, Jihad Mohamad Mansour Abdallah, Patrícia Gaio

**Affiliations:** 1 Instituto de Moléstias Vasculares, Departamento de Cirurgia Vascular, Maringá, PR, Brasil.; 2 Hospital Santa Rita, Departamento de Cirurgia Vascular, Maringá, PR, Brasil.; 3 Hospital Santa Casa de Cianorte, Departamento de Cirurgia Vascular, Cianorte, PR, Brasil.

**Keywords:** dispositivo de oclusão vascular, tempo de hemostasia, compressão manual

## Abstract

**Contexto:**

Os dispositivos de oclusão vascular (DOV) permitem rápida remoção da bainha introdutora de um acesso arterial, reduzindo o tempo de hemostasia, a restrição do paciente ao leito e as complicações no sítio de punção.

**Objetivos:**

Avaliar a eficácia e possíveis complicações do uso de dispositivo de oclusão arterial comparado com a compressão manual.

**Métodos:**

Estudo longitudinal prospectivo randomizado com 20 pacientes no período de dezembro de 2014 a julho de 2015 em Maringá (PR). Foram divididos em dois grupos: aqueles que utilizaram DOV (grupo DOV) e aqueles submetido apenas a compressão manual (grupo CM). Realizaram-se exames de ultrassom Doppler para avaliar a espessura pele-artéria pré e pós-procedimento e verificou-se o tempo de compressão e de deambulação. Os dados foram analisados pelo Programa Statistical Analysis Software.

**Resultados:**

Um total de 60% dos pacientes eram do sexo masculino e a média de idade de ambos os grupos foi de aproximadamente 60 anos. Não houve diferença na espessura pele-artéria entre os grupos. O tempo de compressão no grupo DOV foi de 2 minutos e no grupo CM foi de 21±2,11 minutos (p = 0,0005), e o tempo para retorno de movimentos no membro inferior puncionado foi de 2,35±0,75 horas no grupo DOV e de 6 horas no grupo CM (p = 0,0005). Não houve complicações.

**Conclusões:**

Neste estudo a hemostasia por compressão manual foi tão efetiva quanto o uso de DOV, embora o tempo de compressão e o tempo para retorno às atividades sejam menores nos pacientes submetidos ao uso do dispositivo.

## INTRODUÇÃO

Os dispositivos de oclusão vascular (DOV) foram desenvolvidos para permitir a rápida remoção da bainha introdutora de um acesso arterial após um procedimento endovascular, reduzindo os tempos de hemostasia e a restrição do paciente ao leito e minimizando, em tese, a ocorrência de complicações no sítio de punção. Seu uso limitado é decorrente do aumento de custo que acarreta ao procedimento e da falta de dados demonstrando redução significativa de complicações vasculares em comparação com a compressão manual^1^.

Existem duas categorias de oclusores: os considerados passivos, constituídos de *patch* externo de protrombina ou assistência mecânica de compressão e que não fornecem hemostasia imediata (tempo menor que 5 minutos); e os ativos, que são constituídos por alguma substância ou que realizam a hemostasia através de suturas e fornecem hemostasia imediata^2^.

ExoSeal**^®^** é um dispositivo de oclusão arterial ativo que utiliza o ácido poliglicólico locado em posição extravascular após punção em artéria femoral, fornece hemostasia com menor tempo de compressão e permite deambulação precoce após 2 horas do término do procedimento^2^^,^ 3.

Este estudo tem por objetivo comparar a eficácia e a segurança do dispositivo Exoseal**^®^** Cordis em comparação com a compressão manual.

## MÉTODOS

A amostra deste trabalho é composta por 20 pacientes que foram submetidos a procedimentos diagnósticos ou terapêuticos em cirurgia endovascular no período de dezembro de 2014 a julho de 2015 em dois hospitais (Hospital Santa Rita e Hospital Maringá), ambos situados em Maringá, PR, Brasil. Esses pacientes foram randomizados em dois grupos: pacientes que utilizaram o DOV ExoSeal**^®^** Cordis (grupo DOV) e pacientes submetidos apenas a compressão manual (grupo CM). Trata-se de um estudo longitudinal prospectivo randomizado.

**Figura 1:**
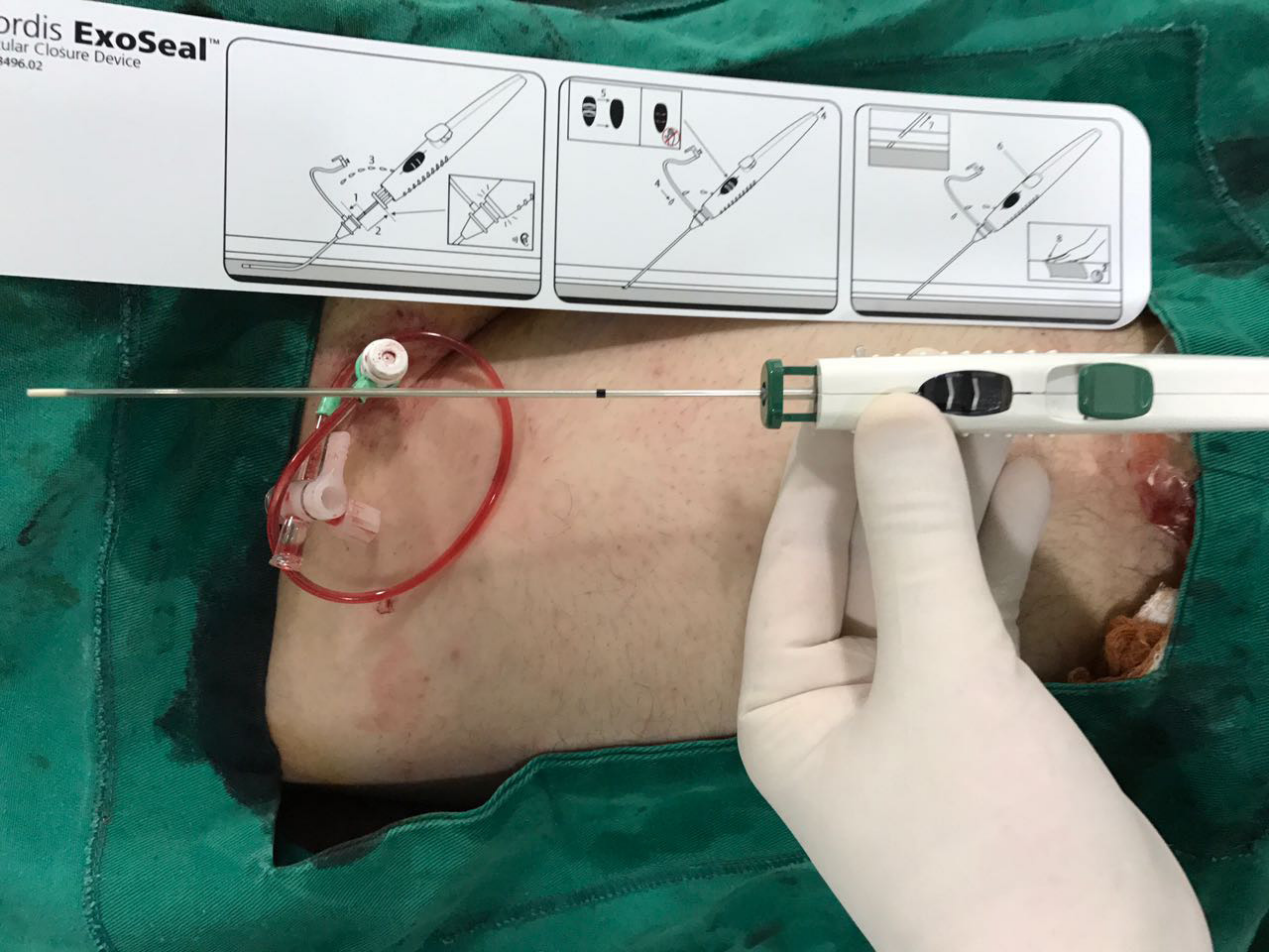
Dispositivo de oclusão vascular.

**Figura 2:**
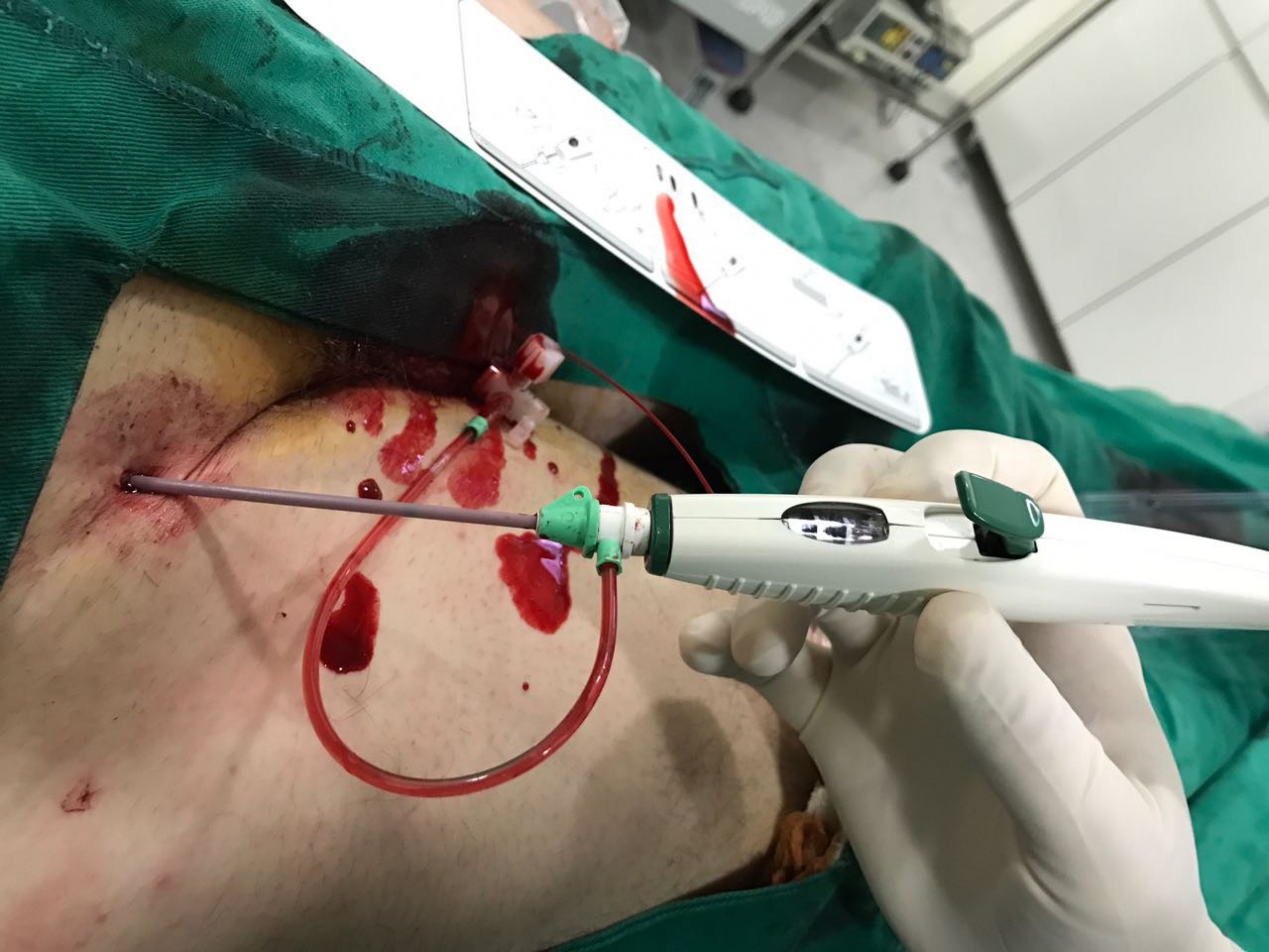
Inserção do dispositivo no introdutor.

O dispositivo é facilmente utilizável inserindo-o no introdutor com sua janela de indicação para cima para a visualização pelo cirurgião. Avança-se o dispositivo até o encontro de seu anel com a válvula hemostática do introdutor, quando se ouve um “clique”, que é comprovado com a saída de sangue pulsátil (*bleed back indicator*) pela lateral. Remove-se o conjunto em ângulo de 30-45° até que o fluxo pulsátil pare e a janela mude a coloração para preto/preto, indicando que o plugue está na posição correta. Nesse momento dispara-se o plugue e a seguir o conjunto (introdutor + ExoSeal**^®^**) é removido em bloco realizando compressão leve no sítio de punção e o curativo é realizado conforme protocolo do serviço^2^^,^ 3 (Figuras 1, 2 e 3).

**Figura 3:**
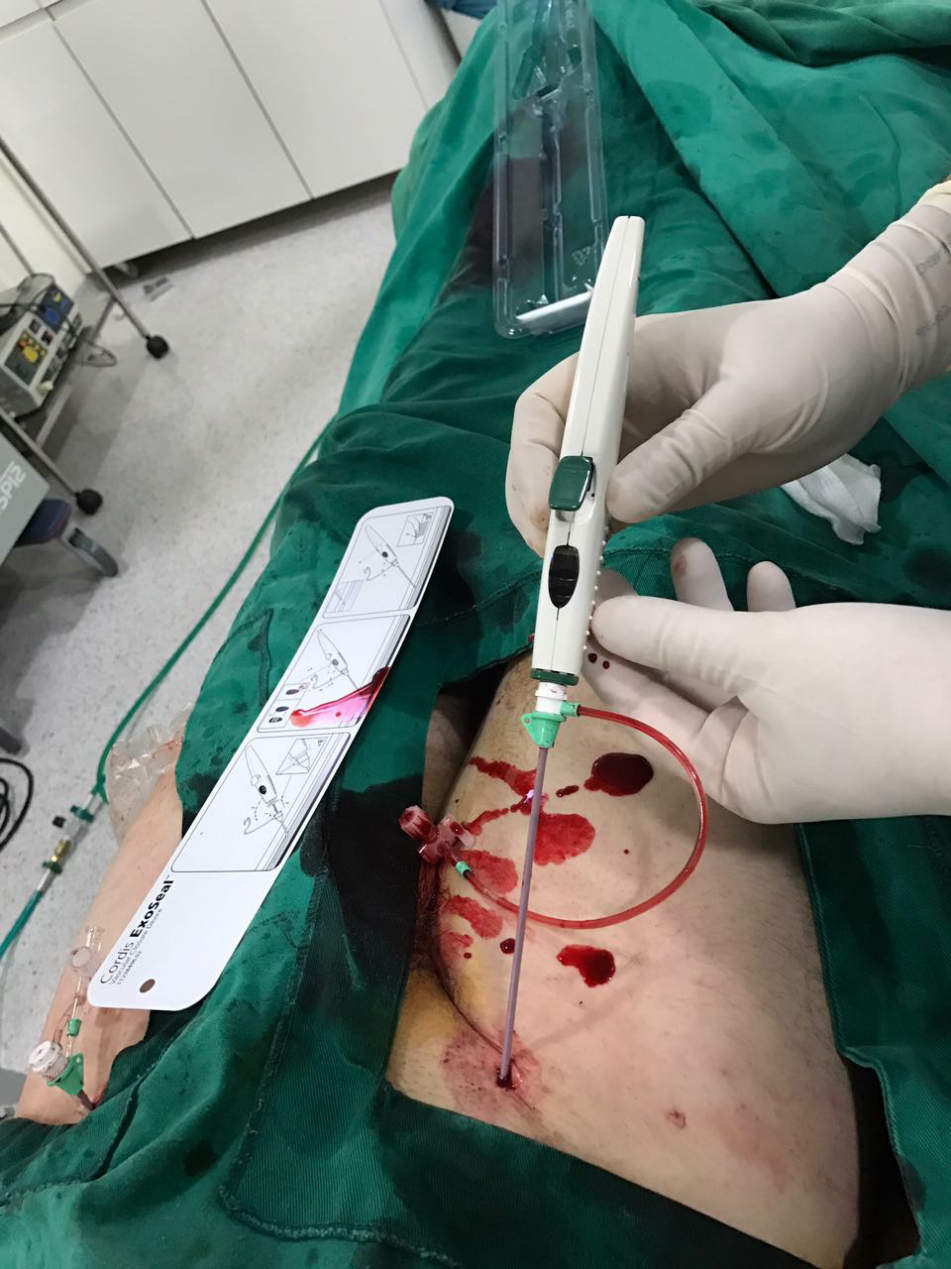
Posicionamento adequado do dispositivo.

Os pacientes foram submetidos a um questionário e a exames de ultrassonografia Doppler arterial pré e pós-procedimento que avaliaram a espessura do tecido subcutâneo entre a pele e artéria, denominada no estudo como “espessura pele-artéria”, e possíveis complicações. Foi verificado o tempo de compressão pós-procedimento e o intervalo de tempo para reiniciar os movimentos do membro inferior puncionado.

Nos pacientes que utilizaram o DOV, após o término do procedimento e remoção do conjunto (introdutor + ExoSeal**^®^**) foi realizada a compressão sobre o sítio de punção pelo tempo de 2 minutos, conforme especificação do produto. Já nos pacientes submetidos apenas a compressão manual foi realizada apenas a compressão com as duas mãos sobre o sítio de punção por um período de 20 minutos. Aqueles pacientes em que não houve a hemostasia com essas medidas iniciais foram submetidos a compressão mecânica por um tempo a mais, de acordo com a quantidade de sangramento encontrada. O tempo de retorno à deambulação foi aferido a partir do término da hemostasia. Observou que alguns pacientes submetidos a bloqueio anestésico raqui/peridural apresentaram um tempo maior de retorno aos movimentos, de acordo com cada tipo de anestesia. A especificação técnica do dispositivo menciona o tempo de 2 horas para o retorno à deambulação, enquanto o nosso serviço utiliza um tempo de 6 horas para os pacientes submetidos a compressão manual.

Após o procedimento, os pacientes submetidos a exames diagnósticos não receberam medicamentos que interferissem nos mecanismos de coagulação. Por outro lado, os pacientes submetidos a angioplastia foram tratados com dupla antiagregação plaquetária com 200 mg de AAS e 150 mg de clopidogrel no pós-operatório imediato.

Foram incluídos no estudo apenas pacientes maiores de 18 anos submetidos a punção retrógrada de artéria femoral com uso de introdutores entre 5 e 7F e que aceitaram participar do estudo através da assinatura do Termo de Consentimento Livre e Esclarecido. Foram excluídos do estudo os pacientes que tinham as artérias puncionadas muito calcificadas, por ser uma das contraindicações do uso do DOV.

Para testar a diferença entre a espessura pele-artéria antes e depois da realização do procedimento e com e sem o uso do dispositivo, foram utilizados os testes de Shapiro-Wilk e de Wilcoxon para verificar a homogeneidade dos grupos. Para análise da significância estatística, foi considerado um nível de confiança de 95% (α = 0,05), ou seja, p-valor menor que 0,05. Os dados foram analisados no programa Statistical Analysis Software (SAS, *version* 9,0), a partir de uma base de dados construída por meio do aplicativo Excel^4^.

## RESULTADOS

A amostra foi composta de oito (40%) pacientes do sexo feminino e 12 (60%) do sexo masculino, com média de idade semelhantes entre os grupos: 60,8±14,5 anos para o grupo DOV e 63,9±16,8 anos para o grupo CM. Foram seis (30%) procedimentos de exame diagnóstico e 14 (70%) procedimentos terapêuticos, e o tempo de procedimento médio foi de 72,0±39,1 minutos para o grupo DOV e 81,0±73,6 minutos para o grupo CM. Não houve falhas e complicações em ambos os grupos estudados. As variáveis de fatores de risco da amostra de pacientes estão descritas na Tabela 1.

**Tabela 1:** Distribuição de frequência dos fatores de risco associados.

	**Sim**	**Não**
Hipertensão arterial	13 (65%)	7 (35%)
Diabetes melito	9 (45%)	11 (55%)
Dislipidemia	8 (40%)	12 (60%)
Tabagismo	12 (60%)	8 (40%)
Obesidade	5 (25%)	15 (75%)
Revascularização miocárdio/angioplastia coronariana	2 (10%)	18 (90%)
Acidente vascular cerebral/ataque isquêmico transitório	3 (15%)	17 (85%)

Para testar a diferença entre a espessura pele-artéria antes e depois, com e sem o uso do dispositivo, foi realizado o teste de Shapiro-Wilk para se verificar a normalidade da variável “espessura pele-artéria” antes de se realizar o procedimento cirúrgico (Tabela 2). A normalidade dos dados foi observada somente para o grupo sem o uso de dispositivo: espessura antes (p=0,0200) e espessura depois (p=0,0407). Para o grupo com uso do dispositivo não foi observada a normalidade dos dados: espessura antes (p=0,9017) e espessura depois (p=0,3392). Por isso, ambos os grupos foram comparados utilizando o teste de Wilcoxon (Tabela 3) para testar se havia diferença significativa entre as espessuras iniciais (antes de se realizar o procedimento) dos pacientes alocados em cada grupo (com e sem dispositivo). O objetivo desse teste foi verificar se a amostra de pacientes era homogênea em relação à variável “espessura pele-artéria” antes de se realizar o procedimento cirúrgico. Não foi observada diferença significativa (p = 0,4265). Assim sendo, a amostra foi considerada homogênea. Na última etapa da análise foi verificado se havia diferença significativa entre as espessuras pele-artéria dos pacientes após a realização do procedimento cirúrgico. Não foi observada diferença entre os grupos (p= 0,4809) conforme mostra a Tabela 4.

**Tabela 2:** Teste de normalidade para a variável distância pele-artéria antes e depois do procedimento cirúrgico nos grupos com e sem dispositivo.

**Espessura**	**Sem uso do dispositivo**		**Com uso do dispositivo**	
	**Antes**	**Depois**	**Antes**	**Depois**
Média	2,0830	2,3820	1,9000	2,258
Desvio-padrão	0,3848	0,3428	0,5375	0,6689
Shapiro-Wilk	0,8115	0,8371	0,9712	0,9178
p-valor	0,0200	0,0407	0,9017	0,3392

**Tabela 3:** Teste de Wilcoxon, para verificar a diferença da distância pele-artéria antes do procedimento cirúrgico nos grupos com e sem dispositivo.

**Uso do dispositivo**	**Escore médio**	**W**	**Aproximação (Z)**	***p*-valor**
Não	11,60	116,00	0,7952	0,4265
Sim	9,40			

**Tabela 4:** Teste de Wilcoxon, para verificar a diferença da distância pele-artéria após procedimento cirúrgico nos grupos com e sem dispositivo.

**Uso do dispositivo**	**Escore médio**	**W**	**Aproximação (Z)**	***p*-valor**
Não	9,50	95,00	-0,7189	0,4809
Sim	11,50			

No quesito tempo de compressão pós-procedimento, ou seja, logo após a retirada do introdutor, todos os pacientes do grupo DOV obtiveram hemostasia adequada após 2 minutos de compressão, enquanto que o grupo CM apresentou um tempo de compressão de 21±2,11 minutos (p = 0,0005). Em relação ao tempo para retorno dos movimentos do membro inferior puncionado, o grupo DOV levou 2,35±0,75 horas, enquanto o grupo CM apresentou tempo de 6 horas (p = 0,0005), demonstrando que os pacientes que utilizaram o dispositivo necessitaram de menor tempo de trabalho do profissional que exercia a compressão e puderam retornar mais precocemente a atividades básicas.

Não houve nenhuma complicação nos pacientes que participaram da pesquisa.

## DISCUSSÃO

ExoSeal**^®^** é um dispositivo percutâneo de oclusão arterial mecânico ativo que utiliza um tampão de ácido poliglicólico bioabsorvível locado em posição extravascular. Esse dispositivo é indicado em punções retrógradas de artéria femoral comum em que se utilizaram introdutores de 5F a 7F, reduzindo o tempo de hemostasia e permitindo uma deambulação precoce (após 2 horas). O uso do ExoSeal**^®^** está contraindicado em artérias muito calcificadas, artérias com diâmetro menor que 5 mm e em pacientes com alergia ao ácido poliglicólico. Complicações podem ocorrer e são limitadas ao sítio de punção, como hematomas, sangramentos, entre outros^5^.

Não encontramos na literatura estudos comparando a espessura entre a pele e a artéria puncionada. Talvez isso não seja mencionado nos estudos por não necessariamente influenciar na presença de complicações como fistulas, pseudoaneurismas, sangramentos maiores, hematomas, entre outros. Em nosso estudo não encontramos diferença estatística nessa mensuração entre o uso ou não do DOV pós-punção arterial.

Quanto à redução do tempo de recuperação e aumento de conforto para os pacientes submetidos a procedimentos endovasculares, não há dúvidas do benefício causado nos pacientes em que foram utilizados os DOV^6^^-^ 10. Nosso estudo comprova os dados da literatura demonstrando que houve diferença importante tanto no menor tempo de compressão exercida pelo profissional sobre o sítio de punção quanto no menor tempo para o retorno da deambulação.

No entanto, ainda não estão estabelecidas as vantagens em termos de efetividade, segurança e custo que justifiquem o uso do dispositivo de modo mais deliberado. A efetividade ou taxa de sucesso, isto é, o percentual de pacientes que atingiram a completa hemostasia com apenas um dispositivo, varia de 87% a 96% e não difere significativamente dos resultados da compressão manual^11^. Em nosso estudo, não houve falhas no grupo que utilizou o dispositivo, o que pode ser justificado pelo pequeno número da amostra.

A incidência de complicações após procedimentos endovasculares é bastante variável, devido às diversas definições encontradas nos estudos e aos inúmeros fatores que contribuem para sua ocorrência (aspecto inerente do paciente, cuidados com a técnica de punção, tamanho e tempo de permanência da bainha introdutora, entre outros)^12^^-^ 15. Devido a essa ampla variedade, os estudos exibem resultados discordantes, desde efeitos protetores a aumento do risco de complicações^16^^-^ 18.

Outro tópico controverso consiste no impacto econômico. Alguns autores demonstraram redução do custo devido à menor estadia hospitalar pós-procedimento e ao menor custo com pessoal requerido para realizar a compressão^19^^,^ 20. Outros estudos já demonstram que essa vantagem acaba sendo anulada pelo elevado custo do DOV utilizado^21^. Trazendo essa discussão para a nossa realidade, Gioppato et al. desenvolveram um estudo que avaliou o custo total somando os valores referentes ao tratamento de complicações (pseudoaneurisma tratado com injeção de trombina guiada por ultrassom Doppler) que ocorreram somente no grupo submetido a compressão manual^11^. Os autores chegaram à conclusão de que, apesar de o custo individual do tratamento das complicações relacionadas à hemostasia por compressão ter sido expressivamente maior, na análise do custo por grupo observa-se que o custo total relativo do grupo que utilizou o DOV foi significativamente maior quando comparado ao do grupo submetido somente a compressão manual^11^.

## CONCLUSÃO

Os resultados observados neste estudo permitem sugerir que a técnica de hemostasia por compressão manual, quando bem realizada, é tão efetiva quanto a hemostasia com os DOV. No entanto, o tempo de compressão exercida pelo profissional e o tempo para retorno à deambulação foram maiores no grupo submetido a compressão manual.

## References

[1] Dauerman HL, Applegate RJ, Cohen DJ (2007). Vascular closure devices: the second decade. J Am Coll Cardiol.

[2] Lobato AC (2015). Cirurgia endovascular.

[3] Exoseal®Vascular Closure Device Angio-seal evolution instructions for use.

[4] Stokes ME, Davis CS, Koch GG (2000). Categorical data analysis using SAS system.

[5] Wong SC, Bachinsky W, Cambier P (2009). A randomized comparison of a novel bioabsorbable vascular closure device versus manual compression in the achievement of hemostasis after percutaneous femoral procedures: the ECLIPSE (Ensure’s Vascular Closure Device Speeds Hemostasis Trial). J Am Coll Cardiol Intv..

[6] Cox T, Blair L, Huntington C, Lincourt A, Sing R, Heniford BT (2015). Systematic review of randomized controlled trials comparing manual compression to vascular closure devices for diagnostic and therapeutic arterial procedures. Surg Technol Int.

[7] Brito FS, Magalhães MA, Nascimento TCDC (2007). Incidência e preditores contemporâneos de complicações vasculares após intervenção coronária percutânea. Rev Bras Cardiol Invasiva..

[8] Duffin DC, Muhlestein JB, Allison SB (2001). Femoral arterial puncture management after percutaneous coronary procedures: a comparison of clinical outcomes and patient satisfaction between manual compression and two different vascular closure devices. J Invasive Cardiol.

[9] Legrand V, Doneux P, Martinez C, Gach O, Bellekens M (2005). Femoral access management: comparison between two different vascular closure devices after percutaneous coronary intervention. Acta Cardiol.

[10] Martin JL, Pratsos A, Magargee E (2008). A randomized trial comparing compression, Perclose Proglide and Angio-Seal VIP for arterial closure following percutaneous coronary intervention: the CAP trial. Catheter Cardiovasc Interv.

[11] Gioppato S, Castello HJ, Conforti TB, Gonçalves SLP, Morais FGS, Cantarelli MJC (2011). Análise da relação custo-efetividade do dispositivo de oclusão vascular AngioSeal^TM^ comparado à compressão manual e/ou mecânica após intervenções endovasculares. Rev Bras Cardiol Invasiva.

[12] Zukowski CN, Costa RJ, Costa R (2010). Preditores e impacto clínico intra-hospitalar do sangramento associado à intervenção coronária percutânea. Rev Bras Cardiol Invasiva..

[13] Bogart DB, Bogart MA, Miller JT, Farrar MW, Barr WK, Montgomery MA (1995). Femoral artery catheterization complications: a study of 503 consecutive patients. Cathet Cardiovasc Diagn.

[14] Piper WD, Malenka DJ, Ryan TJ (2003). Predicting vascular complications in percutaneous coronary interventions. Am Heart J.

[15] Applegate RJ, Sacrinty MT, Kutcher MA (2006). Propensity score analysis of vascular complications after diagnostic cardiac catheterization and percutaneous coronary intervention 1998-2003. Catheter Cardiovasc Interv.

[16] Biancari F, D’Andrea V, Di Marco C, Savino G, Tiozzo V, Catania A (2010). Meta-analysis of randomized trials on the efficacy of vascular closure devices after diagnostic angiography and angioplasty. Am Heart J.

[17] Vaitkus PT (2004). A meta-analysis of percutaneous vascular closure devices after diagnostic catheterization and percutaneous coronary intervention. J Invasive Cardiol.

[18] Koreny M, Riedmüller E, Nikfardjam M, Siostrzonek P, Müllner M (2004). Arterial puncture closing devices compared with standard manual compression after cardiac catheterization: systematic review and meta-analysis. JAMA.

[19] Rickli H, Unterweger M, Sütsch G (2002). Comparison of costs and safety of a suture-mediated closure device with conventional manual compression after coronary artery interventions. Catheter Cardiovasc Interv.

[20] Carere RG, Webb JG, Buller CE (2000). Suture closure of femoral arterial puncture sites after coronary angioplasty followed by same-day discharge. Am Heart J.

[21] Noguchi T, Miyazaki S, Yasuda S (2000). A randomized controlled trial of Prostar Plus for haemostasis in patients after coronary angioplasty. Eur J Vasc Endovasc Surg.

