# Supplementary material for: Avaliação técnica do dispositivo de fechamento vascular Exoseal-Cordis®
Source: J Vasc Bras. 2017 Apr-Jun;16(2):98–103. [Article in Portuguese] doi: 10.1590/1677-5449.000717 (PMC5915857; doi:10.1590/1677-5449.000717)
Supplement: Translation

# Translation: Technical evaluation of Exoseal-Cordis^®^ vascular closure device

**Authors:** Altino Ono Moraes, Rogério Yoshikazu Nabeshima, Ericsson Fernando Viotto, Marcelo Hiroshi Estevam Yoshida, Jihad Mohamad Mansour Abdallah, Patrícia Gaio

**Affiliations:** 1 Instituto de Moléstias Vasculares, Departamento de Cirurgia Vascular, Maringá, PR, Brazil.; 2 Hospital Santa Rita, Departamento de Cirurgia Vascular, Maringá, PR, Brazil.; 3 Hospital Santa Casa de Cianorte, Departamento de Cirurgia Vascular, Cianorte, PR, Brazil.

**Keywords:** vascular closure device, duration of hemostasis, manual compression

## Abstract

**Background:**

Vascular closure devices (VCD) make it possible to rapidly remove the introducer sheath from an arterial access, thereby reducing the length of time in hemostasis, the time patients are restricted to their beds, and the number of puncture site complications.

**Objectives:**

To evaluate the efficacy and possible complications associated with use of an arterial occlusion device compared with manual compression.

**Methods:**

This was a prospective, randomized, longitudinal study of 20 patients conducted from December 2014 to July 2015 in Maringá, PR, Brazil. They were divided into two groups: those who were treated using a VCD (VCD group) and those for whom only manual compression was used (MC group). Doppler ultrasound examination was used to determine skin-artery depth before and after the procedure and the length of time compression was maintained and the delay before mobilization were also recorded. Data were analyzed using the program Statistical Analysis Software.

**Results:**

A total of 60% of the patients were male and the mean age of both groups was approximately 60 years. There was no difference in skin-artery depth between the groups. The duration of compression in the VCD group was 2 minutes and in the MC group it was 21±2.11 minutes (p = 0.0005), while the delay before return to mobility of the lower limb that had been punctured was 2.35±0.75 hours in the VCD group and 6 hours in the MC group (p = 0.0005). There were no complications.

**Conclusions:**

In this study, hemostasis by manual compression exhibited equal efficacy to use of a VCD, but the duration of compression and delay before resumption of activity were shorter in the patients for whom the device was employed.

## INTRODUCTION

Vascular closure devices (VCD) were developed to allow rapid removal of the introducer sheath from an arterial access after endovascular procedures, reducing the duration of hemostasis and reducing the length of time that the patient is restricted to the bed and, theoretically, reducing the rate of complications at the puncture site. Its limited use is a response to the increases in the cost of the procedure with which its sue is associated and the lack of data demonstrating a significant reduction in vascular complications compared with manual compression.^1^

There are two categories of occluders: the first are known as passive devices and this class includes external prothrombin patches and mechanical compression assistance and does not offer immediate hemostasis (in less than 5 minutes); and the second group comprises active methods, in which hemostasis is achieved with some type of substance or with sutures, offering immediate hemostasis.^2^

ExoSeal**^®^** is an active arterial occlusion device that employs a polyglycolic acid plug released in an extravascular position after puncture of the femoral artery, offering hemostasis with shorter duration of compression and enabling early mobilization, 2 hours after completion of the procedure.^2^^,^ 3

The objective of this study is to compare the efficacy and safety of the Exoseal**^®^** Cordis device compared with manual compression.

## METHODS

The study sample comprises 20 patients who underwent diagnostic or therapeutic endovascular surgery procedures between December 2014 and July 2015 at two hospitals (Hospital Santa Rita and Hospital Maringá), both located in Maringá, PR, Brazil. These patients were randomized into one of two groups: patients treated using the ExoSeal**^®^** Cordis VCD (VCD group) and patients who were treated with manual compression alone (MC group). This is a prospective, randomized, longitudinal study.

The device is easy to use, starting by inserting it into the introducer with its indicator window upwards so it is easily viewed by the surgeon. The device is advanced until its ring meets the hemostatic valve of the introducer, when there is an audible “click” and correct coupling is confirmed by pulsing bleed-back via the side. The device and sheath are withdrawn together at an angle of 30-45° until bleed back stops and the indicator window changes from black-white to black-black, indicating that the plug is in the correct position for release. At this point the plug is released and the pair (introducer + ExoSeal**^®^**) of devices are removed together, exerting mild compression on the puncture site, and then a dressing is applied according to the routine protocol used at the service^2^^,^ 3 (Figures 1, 2, and 3).

**Figure 1:** Vascular closure device.

**Figure 2:** Insertion of device in the introducer.

**Figure 3:** Proper positioning of the device.

A questionnaire was administered to the patients and they were examined using Doppler arterial ultrasonography before and after the procedure to determine the thickness of the subcutaneous tissue between the skin and the artery (termed “skin-artery depth” for the purposes of this study) and to detect any possible complications. The duration of compression after the procedure and the interval of time before the punctured lower limb was mobilized once more were both recorded.

For patients treated using the VCD, after the procedure was complete and the introducer + ExoSeal**^®^** pair had been removed, compression was applied over the puncture site for 2 minutes, in accordance with the product’s specifications. For patients in the manual compression group, compression was applied with both hands over the puncture site for 20 minutes. Patients who did not exhibit hemostasis after these measures were subjected to mechanical compression for an additional period, depending on the magnitude of the bleeding observed. The interval until mobilization was measured from the time at which hemostasis was achieved. It was observed that some patients who had been given spinal/peridural anesthetic blockade exhibited a longer duration interval before recovering movement, depending on each type of anesthesia. The technical specifications for the device mention a 2-hour interval for mobilization, while the routine at our service is to allow 6 hours for patients treated using manual compression.

After the procedure, patients who had undergone diagnostic examinations were not given medications that could interfere with coagulation mechanisms. However, patients who had undergone angioplasty were treated with double platelet antiaggregation with 200 mg of aspirin and 150 mg of clopidogrel during the immediate postoperative period.

Only patients over the age of 18 who underwent retrograde puncture of the femoral artery using sheath sizes 5Fr to 7Fr and who agreed to take part, signing a free and informed consent form were enrolled on the study. Patients were excluded from the study if the arteries punctured were highly calcified, because this is one of the contraindications for VCD.

To test the difference between skin-artery depth before and after the procedure and with and without the device, the Shapiro-Wilk and Wilcoxon tests were used to determine homogeneity of groups. Statistical significance was set at a 95% confidence level (α = 0.05), i.e. *p*-values less than 0.05. Data were analyzed using the program Statistical Analysis Software (SAS, version 9.0) and compiled in a database constructed using Excel.^4^

## RESULTS

The sample comprised eight (40%) female patients and 12 (60%) male patients and mean age was similar in both groups: 60.8±14.5 years in the VCD group and 63.9±16.8 years in the MC group. Six (30%) procedures were for diagnostic examinations and 14 (70%) procedures were for treatment, and the mean duration of procedures was 72.0±39.1 minutes for the VCD group and 81.0±73.6 minutes for the MC group. There were no failures or complications in either of the groups studied. The variables for risk factors in the sample of patients are listed in Table 1.

**Table 1:** Frequency distributions of associated risk factors.

	**Yes**	**No**
Arterial hypertension	13 (65%)	7 (35%)
Diabetes mellitus	9 (45%)	11 (55%)
Dyslipidemia	8 (40%)	12 (60%)
Smoking	12 (60%)	8 (40%)
Obesity	5 (25%)	15 (75%)
Myocardial revascularization/coronary angioplasty	2 (10%)	18 (90%)
Stroke/transitory ischemic attack	3 (15%)	17 (85%)

In order to test for differences in skin-artery depth before and after the procedure and with and without the device, the Shapiro-Wilk test was used to verify the normality of the variable “skin-artery depth” before the surgical procedure (Table 2). Data were only normally distributed for the group in which the device was not used: depth before (p=0.0200) and depth after (p=0.0407). In the group in which the device was used, data were not normal: depth before (p=0.9017) and depth after (p=0.3392). In view of this, the results for both groups were compared using the Wilcoxon test (Table 3) to determine whether there were significant difference between baseline thickness (before the procedure) for patients allocated to each group (with and without device). The objective of this test was to test whether the sample of patients was homogeneous in terms of the variable “skin-artery depth” before the surgical procedure. No significant difference was observed (p = 0.4265) and the sample was therefore considered homogeneous. The last step in this analysis was to determine whether there was a significant difference between groups in patients’ skin-artery depth after the surgical procedure. No difference between the groups was observed (p= 0.4809) as shown in Table 4.

**Table 2:** Test of normality for the variable skin-artery depth before and after the surgical procedure for groups treated with and without the device.

**Depth**	**Without device**		**With device**	
	**Before**	**After**	**Before**	**After**
Mean	2.0830	2.3820	1.9000	2.258
Standard deviation	0.3848	0.3428	0.5375	0.6689
Shapiro-Wilk	0.8115	0.8371	0.9712	0.9178
p-value	0.0200	0.0407	0.9017	0.3392

**Table 3:** Wilcoxon test, to analyze difference in skin-artery depth before surgical procedure between groups with and without device.

**Device?**	**Mean score**	**W**	**Approximation (Z)**	***p*-value**
No	11.60	116.00	0.7952	0.4265
Yes	9.40			

**Table 4:** Wilcoxon test, to analyze difference in skin-artery depth after surgical procedure between groups with and without device.

**Device?**	**Mean score**	**W**	**Approximation (Z)**	***p*-value**
No	9.50	95.00	-0.7189	0.4809
Yes	11.50			

With relation to duration of compression after the procedure, i.e., soon after removal of the introducer, all patients in the VCD group exhibited adequate hemostasis after 2 minutes of compression, while duration of compression in the MC group was 21±2.11 minutes (p = 0.0005). With regard to the time taken to mobilize the punctured lower limb, the VCD group took 2.35±0.75 hours, while the MC group had an interval of 6 hours (p = 0.0005), showing that patients with whom the device was used required the professional applying compression to spend less time doing so and were able to resume basic activities more quickly.

There were no complications in any of the patients who took part in this study.

## DISCUSSION

ExoSeal**^®^** is an active percutaneous mechanical device for arterial occlusion that employs a bioabsorbable polyglycolic acid plug that is placed in an extravascular position. The device is indicated for retrograde punctures of the common femoral artery for which 5Fr to 7Fr introducers are used, reducing the time taken to achieve hemostasis and enabling early mobilization (after 2 hours). ExoSeal**^®^** is contraindicated in highly calcified arteries, arteries with diameters of less than 5 mm, and in patients with an allergy to polyglycolic acid. Complications may occur, but are limited to the puncture site, and include hematoma, bleeding, and others.^5^

We were unable to find studies in the literature that compared the distance from the skin to the artery punctured. Perhaps this is not mentioned in studies because it does not necessarily have any influence on complications such as fistulas, pseudoaneurysms, major bleeding, or hematoma, among others. In our study, we did not observe any statistical difference in this measurement after puncture between patients with whom the VCD had or had not been used.

With relation to the reduction in recovery time and increased comfort for patients undergoing endovascular procedures, there is no doubt about the benefits for patients with whom a VCD is used.^6^^-^ 10 Our study confirms already-published data, demonstrating that there was an important difference both in terms of shorter duration of compression exerted by the professional over the puncture site and in terms of shorter time before mobilization.

However, the advantages in terms of efficacy, safety, and cost that would justify use of the device in a more deliberate manner have not yet been established. The efficacy, or success rate, i.e. the percentage of patients for whom complete hemostasis is achieved with just one device, varies from 87% to 96% and does not differ significantly from the results for manual compression.^11^ In our study, there were no failures in the group in which the device was used, which c an be explained by the small sample size.

The incidence of complications after endovascular procedures is highly variable, because of the diversity of definitions used in studies and the countless factors that contribute to their occurrence (aspects inherent to the patients, precautions related to the puncture technique, size and duration of placement of the introducer sheath, and others).^12^^-^ 15 This wide range of variations leads to studies reporting conflicting results that range from protective effects to an increased risk of complications.^16^^-^ 18

Another controversial subject is the financial impact. Some authors have demonstrated cost reductions from a shorter hospital stay after the procedure and reduced expenditure on the personnel required to provide compression.^19^^,^ 20 Other studies have shown that this advantage is wiped out by the high cost of the VCD itself.^21^ Analyzing this debate in our setting (in Brazil), Gioppato et al. conducted a study that investigated the total cost, summing the amounts spent on treatment of complications (pseudoaneurysms treated with Doppler-guided thrombin injections) that only occurred in the group treated using manual compression.^11^ The authors came to the conclusion that, despite the per-individual cost of treatment of complications related to hemostasis by compression being considerably higher, when costs were analyzed by group, the total cost for the VCD group was significantly greater than the cost for the group subjected to manual compression.^11^

## CONCLUSIONS

The results observed in this study allow for the conclusion that the technique of achieving hemostasis by manual compression, when correctly carried out, is equally effective as achieving hemostasis with a VCD. Notwithstanding, duration of compression exerted by the health professional and the time taken to recover mobility were both longer in the group treated using manual compression.
